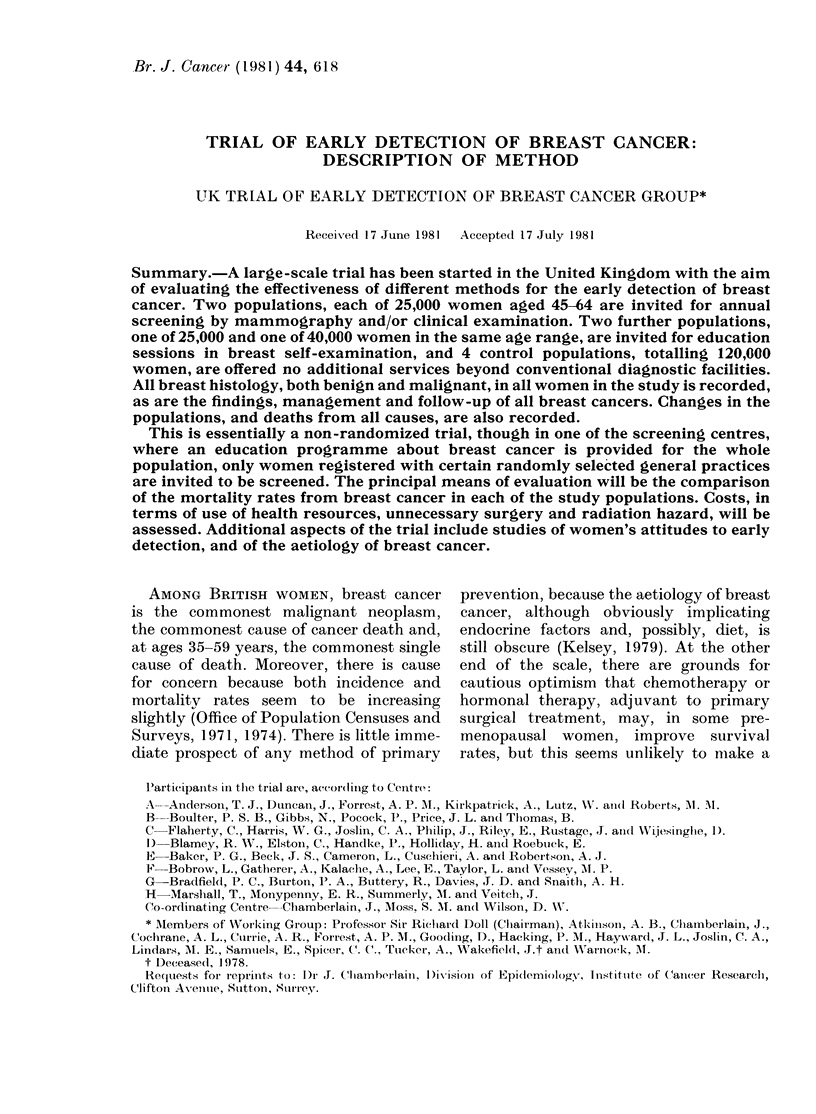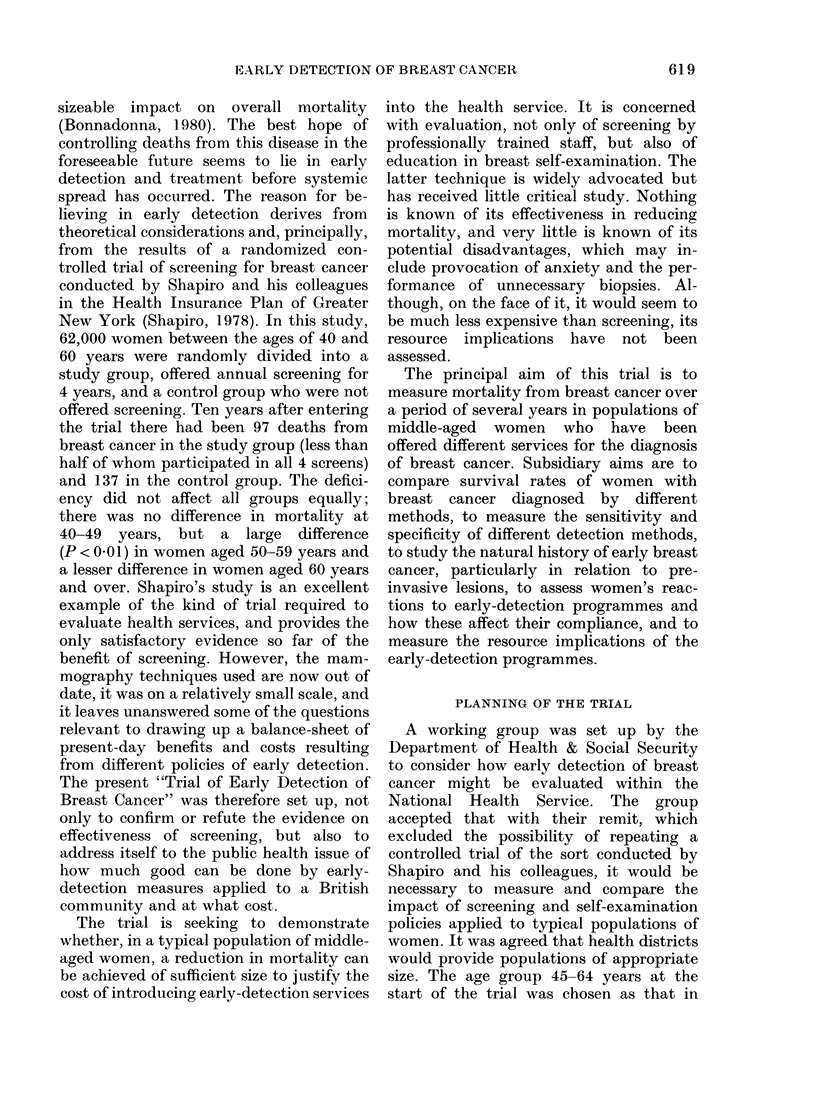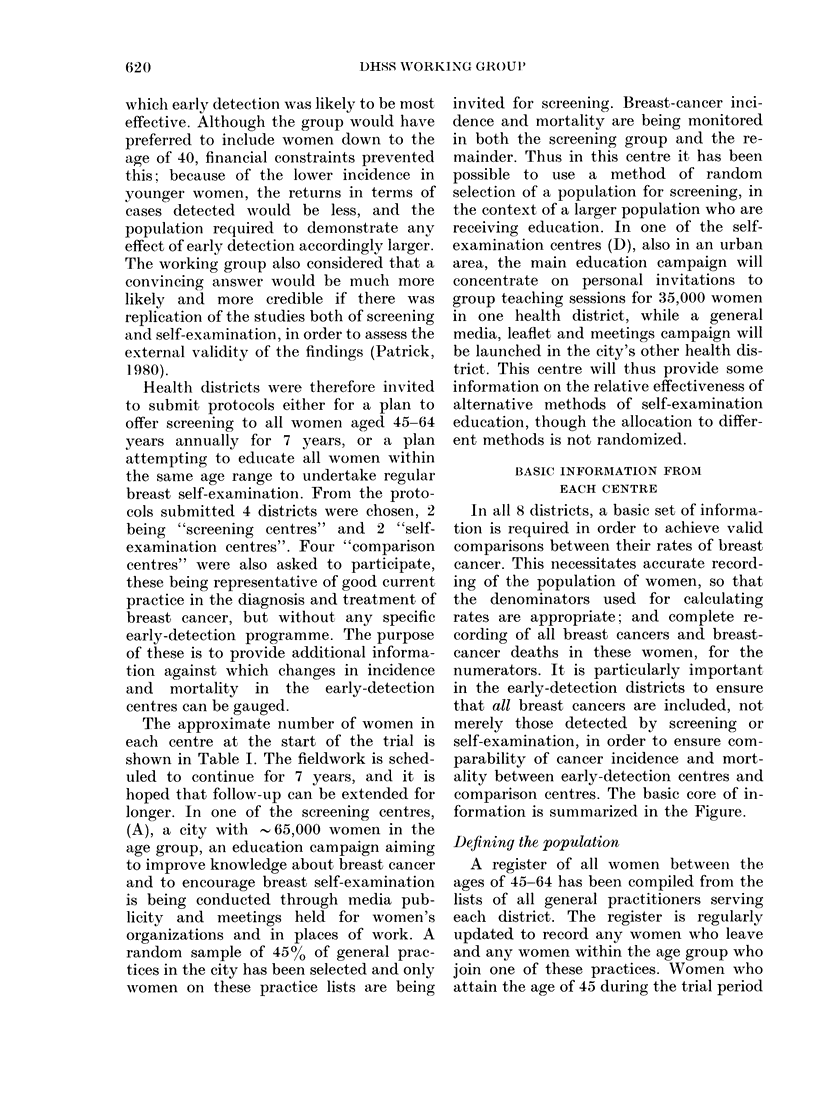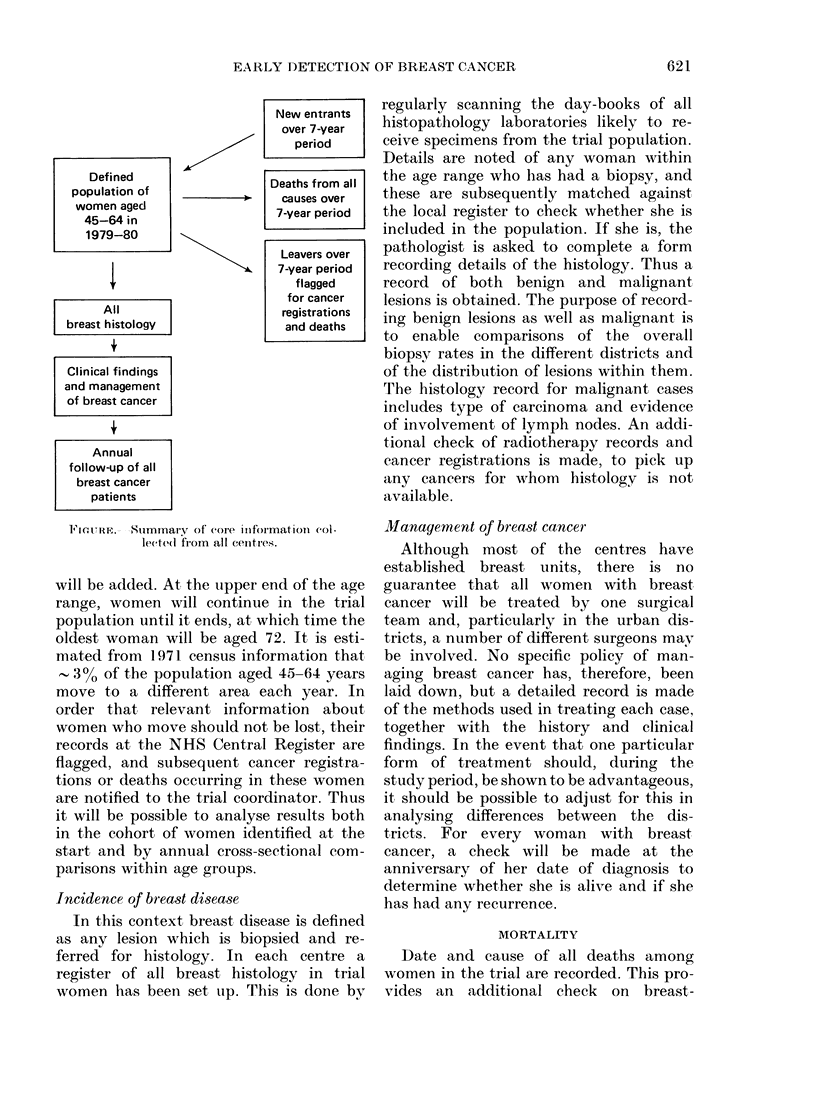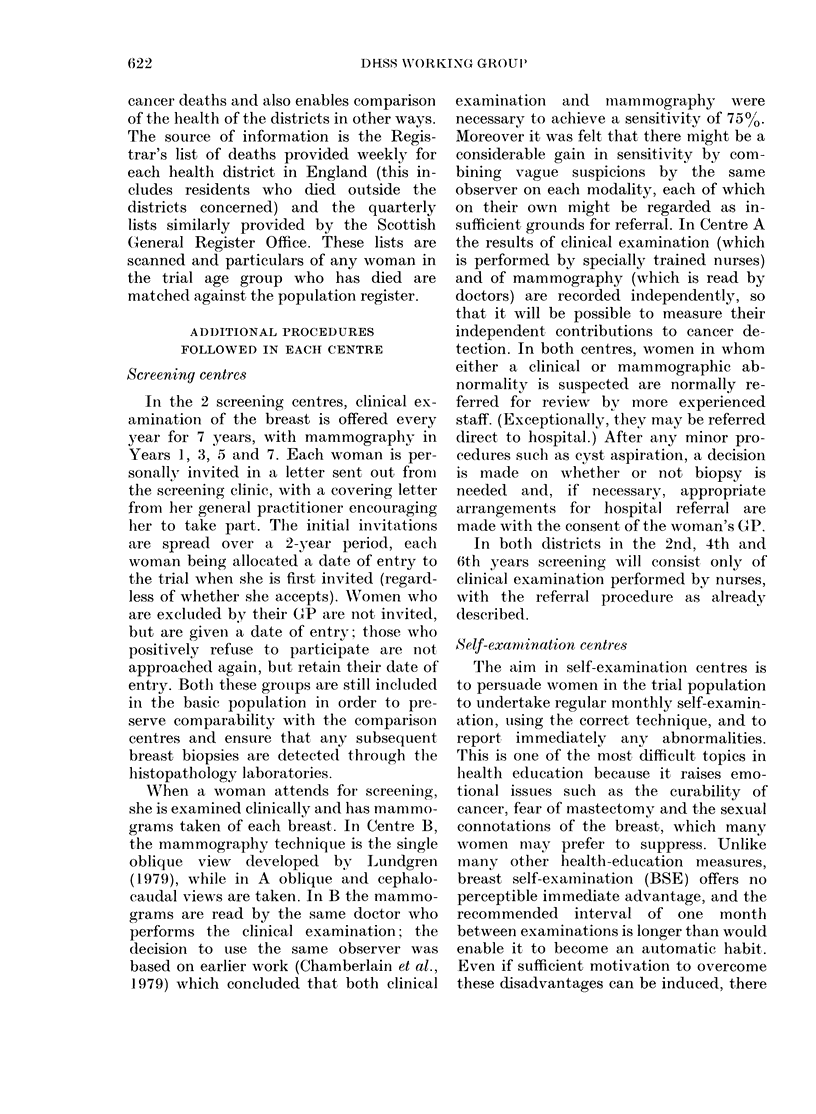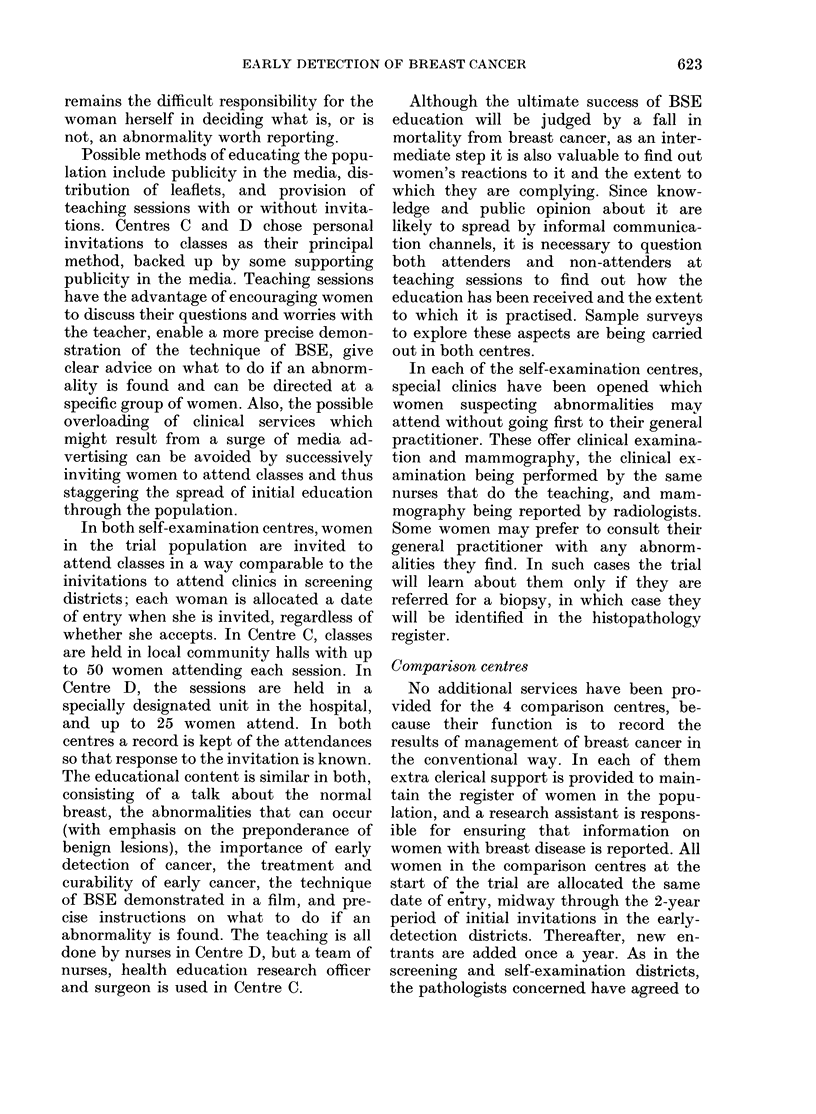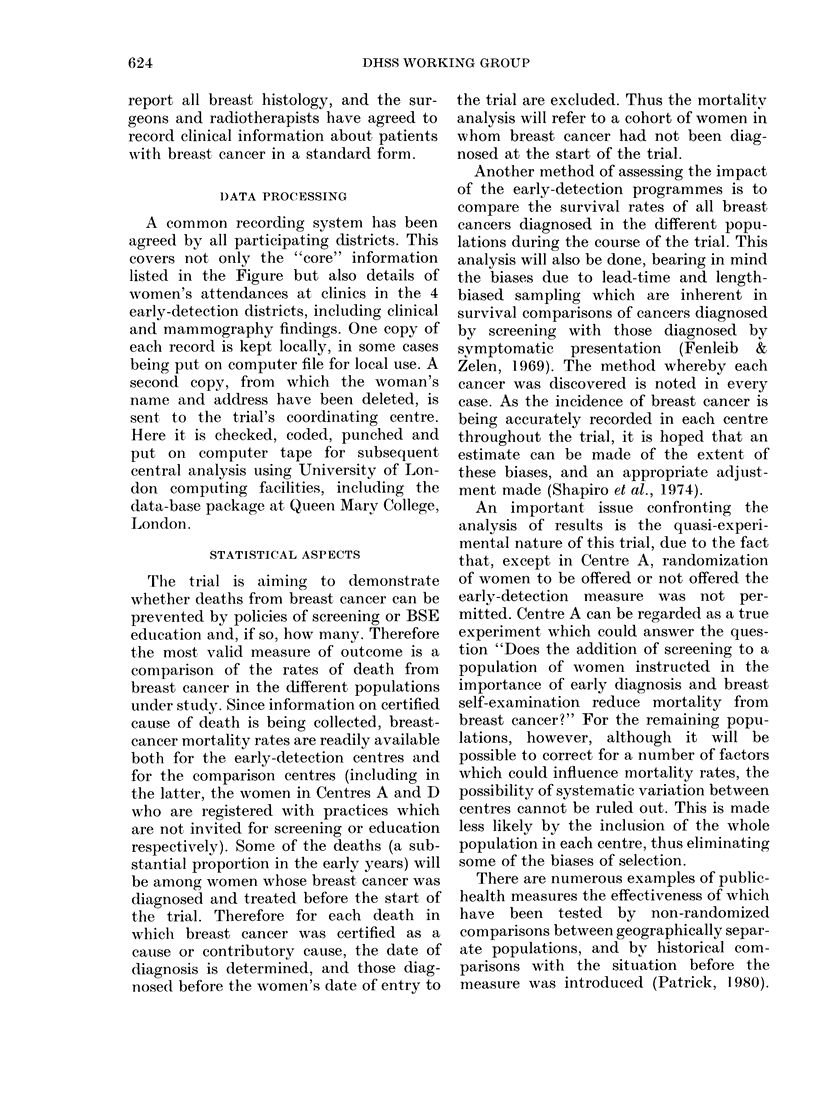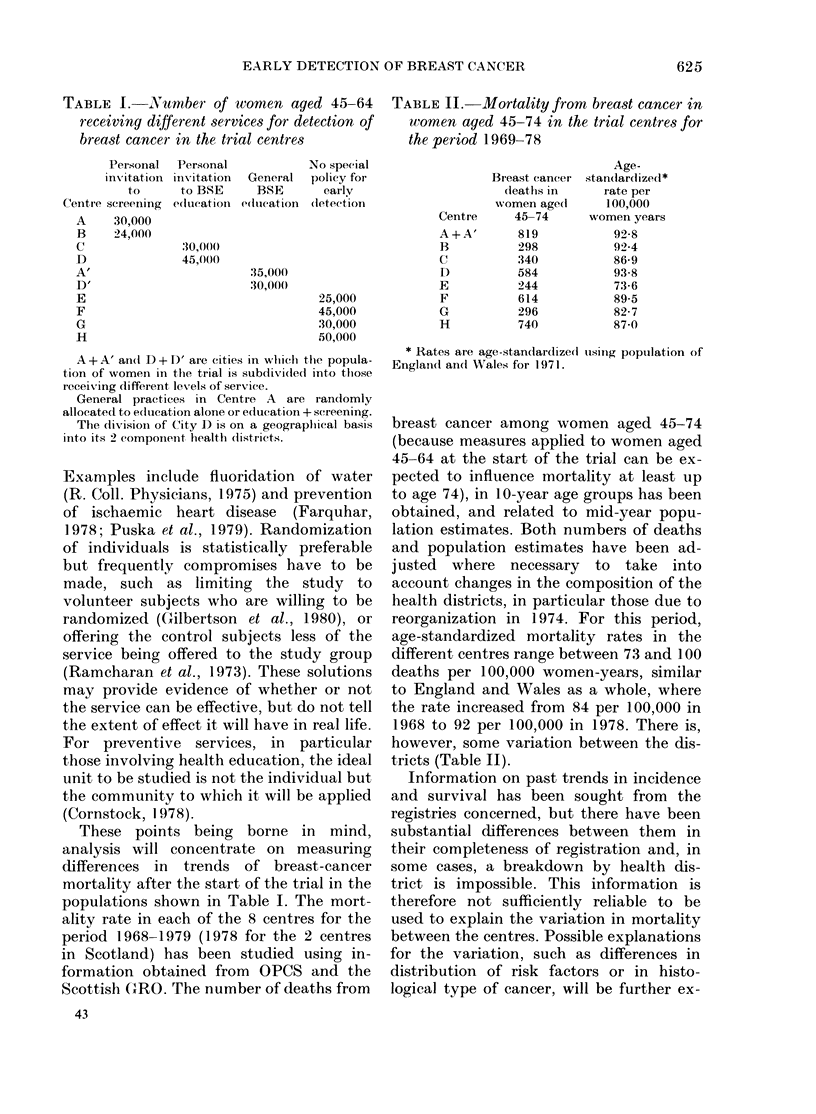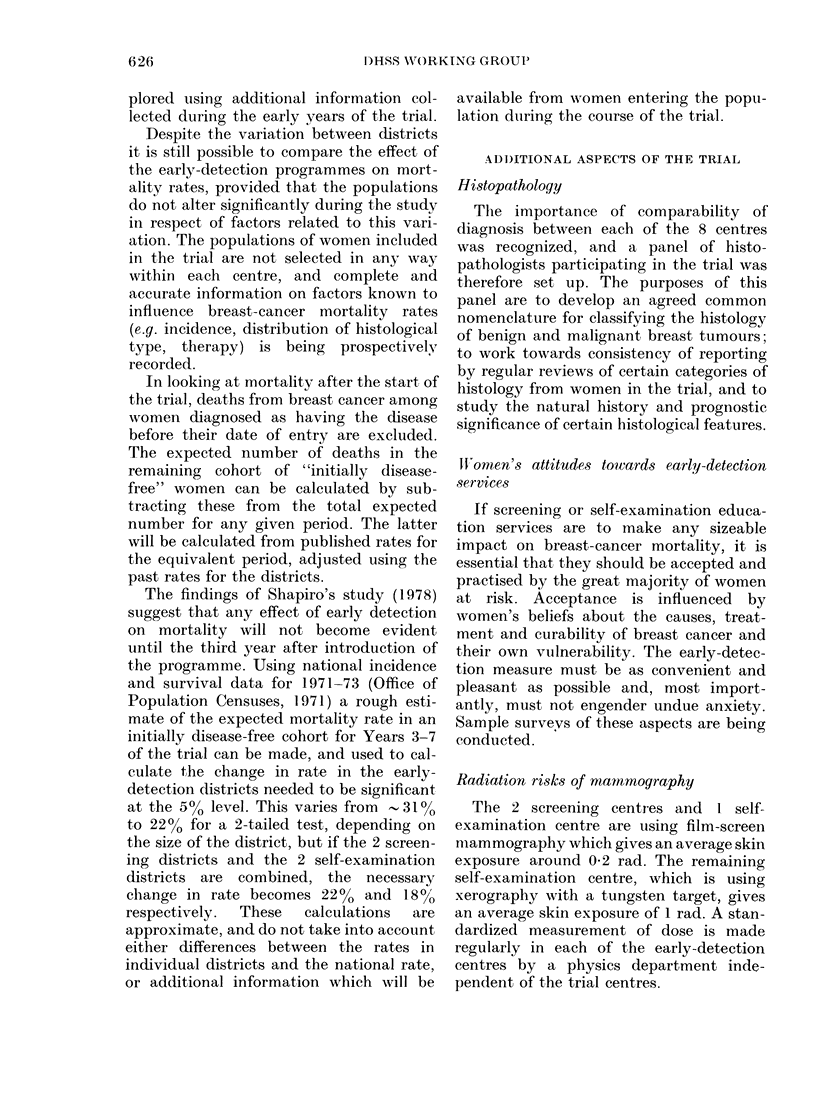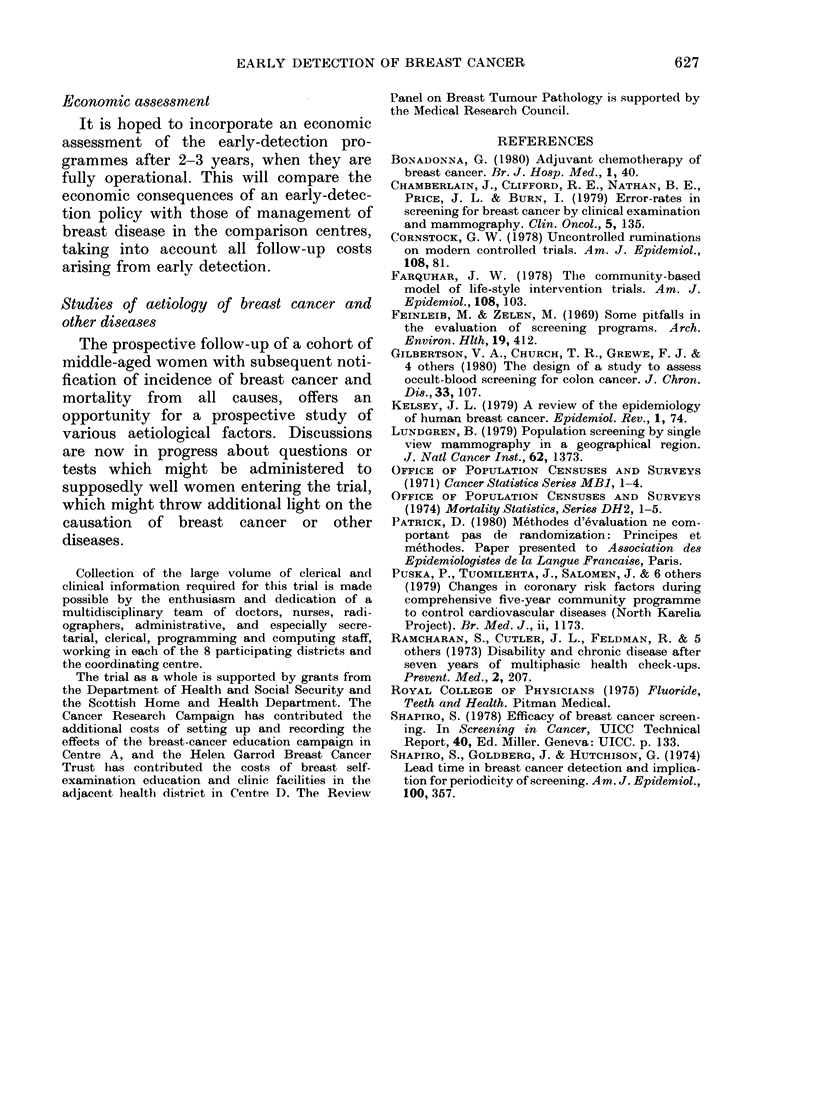# Trial of early detection of breast cancer: description of method.

**DOI:** 10.1038/bjc.1981.246

**Published:** 1981-11

**Authors:** 

## Abstract

A large-scale trial has been started in the United Kingdom with the aim of evaluating the effectiveness of different methods for the early detection of breast cancer. Two populations, each of 25,000 women aged 45-64 are invited for annual screening by mammography and/or clinical examination. Two further populations, one of 25,000 and one of 40,000 women in the same age range, are invited for education sessions in breast self-examination, and 4 control populations, totalling 120,000 women, are offered no additional services beyond conventional diagnostic facilities. All breast histology, both benign and malignant, in all women in the study is recorded, as are the findings, management and follow-up of all breast cancers. Changes in the populations, and deaths from all causes, are also recorded. This is essentially a non-randomized trial, though in one of the screening centres, where an education programme about breast cancer is provided for the whole population, only women registered with certain randomly selected general practices are invited to be screened. The principal means of evaluation will be the comparison of the mortality rates from breast cancer in each of the study populations. Costs, in terms of use of health resources, unnecessary surgery and radiation hazard, will be assessed. Additional aspects of the trial include studies of women's attitudes to early detection, and of the aetiology of breast cancer.


Br. J. Cancer (1981) 44, 618

### TRIAL OF EARLY DETECTION OF BREAST CANCER: DESCRIPTION OF METHOD

UK TRIAL OF EARLY DETECTION OF BREAST CANCER GROUP*

Received 17 June 1981 Accepted 17 July 1981

**Summary.—A large-scale trial has been started in the United Kingdom with the aim of evaluating the effectiveness of different methods for the early detection of breast cancer. Two populations, each of 25,000 women aged 45–64 are invited for annual screening by mammography and/or clinical examination. Two further populations, one of 25,000 and one of 40,000 women in the same age range, are invited for education sessions in breast self-examination, and 4 control populations, totalling 120,000 women, are offered no additional services beyond conventional diagnostic facilities. All breast histology, both benign and malignant, in all women in the study is recorded, as are the findings, management and follow-up of all breast cancers. Changes in the populations, and deaths from all causes, are also recorded.**

**This is essentially a non-randomized trial, though in one of the screening centres, where an education programme about breast cancer is provided for the whole population, only women registered with certain randomly selected general practices are invited to be screened. The principal means of evaluation will be the comparison of the mortality rates from breast cancer in each of the study populations. Costs, in terms of use of health resources, unnecessary surgery and radiation hazard, will be assessed. Additional aspects of the trial include studies of women's attitudes to early detection, and of the aetiology of breast cancer.**

AMONG BRITISH WOMEN, breast cancer is the commonest malignant neoplasm, the commonest cause of cancer death and, at ages 35–59 years, the commonest single cause of death. Moreover, there is cause for concern because both incidence and mortality rates seem to be increasing slightly (Office of Population Censuses and Surveys, 1971, 1974). There is little immediate prospect of any method of primary prevention, because the aetiology of breast cancer, although obviously implicating endocrine factors and, possibly, diet, is still obscure (Kelsey, 1979). At the other end of the scale, there are grounds for cautious optimism that chemotherapy or hormonal therapy, adjuvant to primary surgical treatment, may, in some pre-menopausal women, improve survival rates, but this seems unlikely to make a

Participants in the trial are, according to Centre:

A—Anderson, T. J., Duncan, J., Forrest, A. P. M., Kirkpatrick, A., Lutz, W. and Roberts, M. M.

B—Boulter, P. S. B., Gibbs, N., Pocock, P., Price, J. L. and Thomas, B.

C—Flaherty, C., Harris, W. G., Joslin, C. A., Philip, J., Riley, E., Rustage, J. and Wijesinghe, D.

D—Blamey, R. W., Elston, C., Handke, P., Holliday, H. and Roebuck, E.

E—Baker, P. G., Beck, J. S., Cameron, L., Cuschieri, A. and Robertson, A. J.

F—Bobrow, L., Gatherer, A., Kalache, A., Lee, E., Taylor, L. and Vessey, M. P.

G—Bradfield, P. C., Burton, P. A., Buttery, R., Davies, J. D. and Snaith, A. H.

H—Marshall, T., Monypenny, E. R., Summerly, M. and Veitch, J.

Co-ordinating Centre—Chamberlain, J., Moss, S. M. and Wilson, D. W.

* Members of Working Group: Professor Sir Richard Doll (Chairman), Atkinson, A. B., Chamberlain, J., Cochrane, A. L., Currie, A. R., Forrest, A. P. M., Gooding, D., Hacking, P. M., Hayward, J. L., Joslin, C. A., Lindars, M. E., Samuels, E., Spicer, C. C., Tucker, A., Wakefield, J.† and Warnock, M.

† Deceased, 1978.

Requests for reprints to: Dr J. Chamberlain, Division of Epidemiology, Institute of Cancer Research, Clifton Avenue, Sutton, Surrey.

sizeable impact on overall mortality (Bonnadonna, 1980). The best hope of controlling deaths from this disease in the foreseeable future seems to lie in early detection and treatment before systemic spread has occurred. The reason for believing in early detection derives from theoretical considerations and, principally, from the results of a randomized controlled trial of screening for breast cancer conducted by Shapiro and his colleagues in the Health Insurance Plan of Greater New York (Shapiro, 1978). In this study, 62,000 women between the ages of 40 and 60 years were randomly divided into a study group, offered annual screening for 4 years, and a control group who were not offered screening. Ten years after entering the trial there had been 97 deaths from breast cancer in the study group (less than half of whom participated in all 4 screens) and 137 in the control group. The deficiency did not affect all groups equally; there was no difference in mortality at 40–49 years, but a large difference ($P < 0{\cdot}01$) in women aged 50–59 years and a lesser difference in women aged 60 years and over. Shapiro's study is an excellent example of the kind of trial required to evaluate health services, and provides the only satisfactory evidence so far of the benefit of screening. However, the mammography techniques used are now out of date, it was on a relatively small scale, and it leaves unanswered some of the questions relevant to drawing up a balance-sheet of present-day benefits and costs resulting from different policies of early detection. The present "Trial of Early Detection of Breast Cancer" was therefore set up, not only to confirm or refute the evidence on effectiveness of screening, but also to address itself to the public health issue of how much good can be done by early-detection measures applied to a British community and at what cost.

The trial is seeking to demonstrate whether, in a typical population of middle-aged women, a reduction in mortality can be achieved of sufficient size to justify the cost of introducing early-detection services into the health service. It is concerned with evaluation, not only of screening by professionally trained staff, but also of education in breast self-examination. The latter technique is widely advocated but has received little critical study. Nothing is known of its effectiveness in reducing mortality, and very little is known of its potential disadvantages, which may include provocation of anxiety and the performance of unnecessary biopsies. Although, on the face of it, it would seem to be much less expensive than screening, its resource implications have not been assessed.

The principal aim of this trial is to measure mortality from breast cancer over a period of several years in populations of middle-aged women who have been offered different services for the diagnosis of breast cancer. Subsidiary aims are to compare survival rates of women with breast cancer diagnosed by different methods, to measure the sensitivity and specificity of different detection methods, to study the natural history of early breast cancer, particularly in relation to pre-invasive lesions, to assess women's reactions to early-detection programmes and how these affect their compliance, and to measure the resource implications of the early-detection programmes.

### PLANNING OF THE TRIAL

A working group was set up by the Department of Health & Social Security to consider how early detection of breast cancer might be evaluated within the National Health Service. The group accepted that with their remit, which excluded the possibility of repeating a controlled trial of the sort conducted by Shapiro and his colleagues, it would be necessary to measure and compare the impact of screening and self-examination policies applied to typical populations of women. It was agreed that health districts would provide populations of appropriate size. The age group 45–64 years at the start of the trial was chosen as that in which early detection was likely to be most effective. Although the group would have preferred to include women down to the age of 40, financial constraints prevented this; because of the lower incidence in younger women, the returns in terms of cases detected would be less, and the population required to demonstrate any effect of early detection accordingly larger. The working group also considered that a convincing answer would be much more likely and more credible if there was replication of the studies both of screening and self-examination, in order to assess the external validity of the findings (Patrick, 1980).

Health districts were therefore invited to submit protocols either for a plan to offer screening to all women aged 45–64 years annually for 7 years, or a plan attempting to educate all women within the same age range to undertake regular breast self-examination. From the protocols submitted 4 districts were chosen, 2 being "screening centres" and 2 "self-examination centres". Four "comparison centres" were also asked to participate, these being representative of good current practice in the diagnosis and treatment of breast cancer, but without any specific early-detection programme. The purpose of these is to provide additional information against which changes in incidence and mortality in the early-detection centres can be gauged.

The approximate number of women in each centre at the start of the trial is shown in Table I. The fieldwork is scheduled to continue for 7 years, and it is hoped that follow-up can be extended for longer. In one of the screening centres, (A), a city with ~65,000 women in the age group, an education campaign aiming to improve knowledge about breast cancer and to encourage breast self-examination is being conducted through media publicity and meetings held for women's organizations and in places of work. A random sample of 45% of general practices in the city has been selected and only women on these practice lists are being invited for screening. Breast-cancer incidence and mortality are being monitored in both the screening group and the remainder. Thus in this centre it has been possible to use a method of random selection of a population for screening, in the context of a larger population who are receiving education. In one of the self-examination centres (D), also in an urban area, the main education campaign will concentrate on personal invitations to group teaching sessions for 35,000 women in one health district, while a general media, leaflet and meetings campaign will be launched in the city's other health district. This centre will thus provide some information on the relative effectiveness of alternative methods of self-examination education, though the allocation to different methods is not randomized.

### BASIC INFORMATION FROM EACH CENTRE

In all 8 districts, a basic set of information is required in order to achieve valid comparisons between their rates of breast cancer. This necessitates accurate recording of the population of women, so that the denominators used for calculating rates are appropriate; and complete recording of all breast cancers and breast-cancer deaths in these women, for the numerators. It is particularly important in the early-detection districts to ensure that *all* breast cancers are included, not merely those detected by screening or self-examination, in order to ensure comparability of cancer incidence and mortality between early-detection centres and comparison centres. The basic core of information is summarized in the Figure.

### *Defining the population*

A register of all women between the ages of 45–64 has been compiled from the lists of all general practitioners serving each district. The register is regularly updated to record any women who leave and any women within the age group who join one of these practices. Women who attain the age of 45 during the trial period

FIGURE.—Summary of core information collected from all centres.

will be added. At the upper end of the age range, women will continue in the trial population until it ends, at which time the oldest woman will be aged 72. It is estimated from 1971 census information that $\sim 3\%$ of the population aged 45–64 years move to a different area each year. In order that relevant information about women who move should not be lost, their records at the NHS Central Register are flagged, and subsequent cancer registrations or deaths occurring in these women are notified to the trial coordinator. Thus it will be possible to analyse results both in the cohort of women identified at the start and by annual cross-sectional comparisons within age groups.

*Incidence of breast disease*

In this context breast disease is defined as any lesion which is biopsied and referred for histology. In each centre a register of all breast histology in trial women has been set up. This is done by regularly scanning the day-books of all histopathology laboratories likely to receive specimens from the trial population. Details are noted of any woman within the age range who has had a biopsy, and these are subsequently matched against the local register to check whether she is included in the population. If she is, the pathologist is asked to complete a form recording details of the histology. Thus a record of both benign and malignant lesions is obtained. The purpose of recording benign lesions as well as malignant is to enable comparisons of the overall biopsy rates in the different districts and of the distribution of lesions within them. The histology record for malignant cases includes type of carcinoma and evidence of involvement of lymph nodes. An additional check of radiotherapy records and cancer registrations is made, to pick up any cancers for whom histology is not available.

*Management of breast cancer*

Although most of the centres have established breast units, there is no guarantee that all women with breast cancer will be treated by one surgical team and, particularly in the urban districts, a number of different surgeons may be involved. No specific policy of managing breast cancer has, therefore, been laid down, but a detailed record is made of the methods used in treating each case, together with the history and clinical findings. In the event that one particular form of treatment should, during the study period, be shown to be advantageous, it should be possible to adjust for this in analysing differences between the districts. For every woman with breast cancer, a check will be made at the anniversary of her date of diagnosis to determine whether she is alive and if she has had any recurrence.

### MORTALITY

Date and cause of all deaths among women in the trial are recorded. This provides an additional check on breastcancer deaths and also enables comparison of the health of the districts in other ways. The source of information is the Registrar's list of deaths provided weekly for each health district in England (this includes residents who died outside the districts concerned) and the quarterly lists similarly provided by the Scottish General Register Office. These lists are scanned and particulars of any woman in the trial age group who has died are matched against the population register.

### ADDITIONAL PROCEDURES FOLLOWED IN EACH CENTRE

### *Screening centres*

In the 2 screening centres, clinical examination of the breast is offered every year for 7 years, with mammography in Years 1, 3, 5 and 7. Each woman is personally invited in a letter sent out from the screening clinic, with a covering letter from her general practitioner encouraging her to take part. The initial invitations are spread over a 2-year period, each woman being allocated a date of entry to the trial when she is first invited (regardless of whether she accepts). Women who are excluded by their GP are not invited, but are given a date of entry; those who positively refuse to participate are not approached again, but retain their date of entry. Both these groups are still included in the basic population in order to preserve comparability with the comparison centres and ensure that any subsequent breast biopsies are detected through the histopathology laboratories.

When a woman attends for screening, she is examined clinically and has mammograms taken of each breast. In Centre B, the mammography technique is the single oblique view developed by Lundgren (1979), while in A oblique and cephalocaudal views are taken. In B the mammograms are read by the same doctor who performs the clinical examination; the decision to use the same observer was based on earlier work (Chamberlain *et al.*, 1979) which concluded that both clinical examination and mammography were necessary to achieve a sensitivity of 75%. Moreover it was felt that there might be a considerable gain in sensitivity by combining vague suspicions by the same observer on each modality, each of which on their own might be regarded as insufficient grounds for referral. In Centre A the results of clinical examination (which is performed by specially trained nurses) and of mammography (which is read by doctors) are recorded independently, so that it will be possible to measure their independent contributions to cancer detection. In both centres, women in whom either a clinical or mammographic abnormality is suspected are normally referred for review by more experienced staff. (Exceptionally, they may be referred direct to hospital.) After any minor procedures such as cyst aspiration, a decision is made on whether or not biopsy is needed and, if necessary, appropriate arrangements for hospital referral are made with the consent of the woman's GP.

In both districts in the 2nd, 4th and 6th years screening will consist only of clinical examination performed by nurses, with the referral procedure as already described.

### *Self-examination centres*

The aim in self-examination centres is to persuade women in the trial population to undertake regular monthly self-examination, using the correct technique, and to report immediately any abnormalities. This is one of the most difficult topics in health education because it raises emotional issues such as the curability of cancer, fear of mastectomy and the sexual connotations of the breast, which many women may prefer to suppress. Unlike many other health-education measures, breast self-examination (BSE) offers no perceptible immediate advantage, and the recommended interval of one month between examinations is longer than would enable it to become an automatic habit. Even if sufficient motivation to overcome these disadvantages can be induced, there remains the difficult responsibility for the woman herself in deciding what is, or is not, an abnormality worth reporting.

Possible methods of educating the population include publicity in the media, distribution of leaflets, and provision of teaching sessions with or without invitations. Centres C and D chose personal invitations to classes as their principal method, backed up by some supporting publicity in the media. Teaching sessions have the advantage of encouraging women to discuss their questions and worries with the teacher, enable a more precise demonstration of the technique of BSE, give clear advice on what to do if an abnormality is found and can be directed at a specific group of women. Also, the possible overloading of clinical services which might result from a surge of media advertising can be avoided by successively inviting women to attend classes and thus staggering the spread of initial education through the population.

In both self-examination centres, women in the trial population are invited to attend classes in a way comparable to the inivitations to attend clinics in screening districts; each woman is allocated a date of entry when she is invited, regardless of whether she accepts. In Centre C, classes are held in local community halls with up to 50 women attending each session. In Centre D, the sessions are held in a specially designated unit in the hospital, and up to 25 women attend. In both centres a record is kept of the attendances so that response to the invitation is known. The educational content is similar in both, consisting of a talk about the normal breast, the abnormalities that can occur (with emphasis on the preponderance of benign lesions), the importance of early detection of cancer, the treatment and curability of early cancer, the technique of BSE demonstrated in a film, and precise instructions on what to do if an abnormality is found. The teaching is all done by nurses in Centre D, but a team of nurses, health education research officer and surgeon is used in Centre C.

Although the ultimate success of BSE education will be judged by a fall in mortality from breast cancer, as an intermediate step it is also valuable to find out women's reactions to it and the extent to which they are complying. Since knowledge and public opinion about it are likely to spread by informal communication channels, it is necessary to question both attenders and non-attenders at teaching sessions to find out how the education has been received and the extent to which it is practised. Sample surveys to explore these aspects are being carried out in both centres.

In each of the self-examination centres, special clinics have been opened which women suspecting abnormalities may attend without going first to their general practitioner. These offer clinical examination and mammography, the clinical examination being performed by the same nurses that do the teaching, and mammography being reported by radiologists. Some women may prefer to consult their general practitioner with any abnormalities they find. In such cases the trial will learn about them only if they are referred for a biopsy, in which case they will be identified in the histopathology register.

*Comparison centres*

No additional services have been provided for the 4 comparison centres, because their function is to record the results of management of breast cancer in the conventional way. In each of them extra clerical support is provided to maintain the register of women in the population, and a research assistant is responsible for ensuring that information on women with breast disease is reported. All women in the comparison centres at the start of the trial are allocated the same date of entry, midway through the 2-year period of initial invitations in the early-detection districts. Thereafter, new entrants are added once a year. As in the screening and self-examination districts, the pathologists concerned have agreed to report all breast histology, and the surgeons and radiotherapists have agreed to record clinical information about patients with breast cancer in a standard form.

### DATA PROCESSING

A common recording system has been agreed by all participating districts. This covers not only the "core" information listed in the Figure but also details of women's attendances at clinics in the 4 early-detection districts, including clinical and mammography findings. One copy of each record is kept locally, in some cases being put on computer file for local use. A second copy, from which the woman's name and address have been deleted, is sent to the trial's coordinating centre. Here it is checked, coded, punched and put on computer tape for subsequent central analysis using University of London computing facilities, including the data-base package at Queen Mary College, London.

### STATISTICAL ASPECTS

The trial is aiming to demonstrate whether deaths from breast cancer can be prevented by policies of screening or BSE education and, if so, how many. Therefore the most valid measure of outcome is a comparison of the rates of death from breast cancer in the different populations under study. Since information on certified cause of death is being collected, breast-cancer mortality rates are readily available both for the early-detection centres and for the comparison centres (including in the latter, the women in Centres A and D who are registered with practices which are not invited for screening or education respectively). Some of the deaths (a substantial proportion in the early years) will be among women whose breast cancer was diagnosed and treated before the start of the trial. Therefore for each death in which breast cancer was certified as a cause or contributory cause, the date of diagnosis is determined, and those diagnosed before the women's date of entry to the trial are excluded. Thus the mortality analysis will refer to a cohort of women in whom breast cancer had not been diagnosed at the start of the trial.

Another method of assessing the impact of the early-detection programmes is to compare the survival rates of all breast cancers diagnosed in the different populations during the course of the trial. This analysis will also be done, bearing in mind the biases due to lead-time and length-biased sampling which are inherent in survival comparisons of cancers diagnosed by screening with those diagnosed by symptomatic presentation (Fenleib & Zelen, 1969). The method whereby each cancer was discovered is noted in every case. As the incidence of breast cancer is being accurately recorded in each centre throughout the trial, it is hoped that an estimate can be made of the extent of these biases, and an appropriate adjustment made (Shapiro *et al.*, 1974).

An important issue confronting the analysis of results is the quasi-experimental nature of this trial, due to the fact that, except in Centre A, randomization of women to be offered or not offered the early-detection measure was not permitted. Centre A can be regarded as a true experiment which could answer the question "Does the addition of screening to a population of women instructed in the importance of early diagnosis and breast self-examination reduce mortality from breast cancer?" For the remaining populations, however, although it will be possible to correct for a number of factors which could influence mortality rates, the possibility of systematic variation between centres cannot be ruled out. This is made less likely by the inclusion of the whole population in each centre, thus eliminating some of the biases of selection.

There are numerous examples of public-health measures the effectiveness of which have been tested by non-randomized comparisons between geographically separate populations, and by historical comparisons with the situation before the measure was introduced (Patrick, 1980).

TABLE I.—*Number of women aged 45–64 receiving different services for detection of breast cancer in the trial centres*

| Centre | Personal invitation to screening | Personal invitation to BSE education | General BSE education | No special policy for early detection |
|---|---|---|---|---|
| A | 30,000 | | | |
| B | 24,000 | | | |
| C | | 30,000 | | |
| D | | 45,000 | | |
| A′ | | | 35,000 | |
| D′ | | | 30,000 | |
| E | | | | 25,000 |
| F | | | | 45,000 |
| G | | | | 30,000 |
| H | | | | 50,000 |

A + A′ and D + D′ are cities in which the population of women in the trial is subdivided into those receiving different levels of service.

General practices in Centre A are randomly allocated to education alone or education + screening.

The division of City D is on a geographical basis into its 2 component health districts.

TABLE II.—*Mortality from breast cancer in women aged* 45–74 *in the trial centres for the period* 1969–78

| Centre | Breast cancer deaths in women aged 45–74 | Age-standardized* rate per 100,000 women years |
|---|---|---|
| A + A′ | 819 | 92·8 |
| B | 298 | 92·4 |
| C | 340 | 86·9 |
| D | 584 | 93·8 |
| E | 244 | 73·6 |
| F | 614 | 89·5 |
| G | 296 | 82·7 |
| H | 740 | 87·0 |

* Rates are age-standardized using population of England and Wales for 1971.

Examples include fluoridation of water (R. Coll. Physicians, 1975) and prevention of ischaemic heart disease (Farquhar, 1978; Puska *et al.*, 1979). Randomization of individuals is statistically preferable but frequently compromises have to be made, such as limiting the study to volunteer subjects who are willing to be randomized (Gilbertson *et al.*, 1980), or offering the control subjects less of the service being offered to the study group (Ramcharan *et al.*, 1973). These solutions may provide evidence of whether or not the service can be effective, but do not tell the extent of effect it will have in real life. For preventive services, in particular those involving health education, the ideal unit to be studied is not the individual but the community to which it will be applied (Cornstock, 1978).

These points being borne in mind, analysis will concentrate on measuring differences in trends of breast-cancer mortality after the start of the trial in the populations shown in Table I. The mortality rate in each of the 8 centres for the period 1968–1979 (1978 for the 2 centres in Scotland) has been studied using information obtained from OPCS and the Scottish GRO. The number of deaths from breast cancer among women aged 45–74 (because measures applied to women aged 45–64 at the start of the trial can be expected to influence mortality at least up to age 74), in 10-year age groups has been obtained, and related to mid-year population estimates. Both numbers of deaths and population estimates have been adjusted where necessary to take into account changes in the composition of the health districts, in particular those due to reorganization in 1974. For this period, age-standardized mortality rates in the different centres range between 73 and 100 deaths per 100,000 women-years, similar to England and Wales as a whole, where the rate increased from 84 per 100,000 in 1968 to 92 per 100,000 in 1978. There is, however, some variation between the districts (Table II).

Information on past trends in incidence and survival has been sought from the registries concerned, but there have been substantial differences between them in their completeness of registration and, in some cases, a breakdown by health district is impossible. This information is therefore not sufficiently reliable to be used to explain the variation in mortality between the centres. Possible explanations for the variation, such as differences in distribution of risk factors or in histological type of cancer, will be further explored using additional information collected during the early years of the trial.

Despite the variation between districts it is still possible to compare the effect of the early-detection programmes on mortality rates, provided that the populations do not alter significantly during the study in respect of factors related to this variation. The populations of women included in the trial are not selected in any way within each centre, and complete and accurate information on factors known to influence breast-cancer mortality rates (*e.g.* incidence, distribution of histological type, therapy) is being prospectively recorded.

In looking at mortality after the start of the trial, deaths from breast cancer among women diagnosed as having the disease before their date of entry are excluded. The expected number of deaths in the remaining cohort of "initially disease-free" women can be calculated by subtracting these from the total expected number for any given period. The latter will be calculated from published rates for the equivalent period, adjusted using the past rates for the districts.

The findings of Shapiro's study (1978) suggest that any effect of early detection on mortality will not become evident until the third year after introduction of the programme. Using national incidence and survival data for 1971–73 (Office of Population Censuses, 1971) a rough estimate of the expected mortality rate in an initially disease-free cohort for Years 3–7 of the trial can be made, and used to calculate the change in rate in the early-detection districts needed to be significant at the 5% level. This varies from ~31% to 22% for a 2-tailed test, depending on the size of the district, but if the 2 screening districts and the 2 self-examination districts are combined, the necessary change in rate becomes 22% and 18% respectively. These calculations are approximate, and do not take into account either differences between the rates in individual districts and the national rate, or additional information which will be available from women entering the population during the course of the trial.

### ADDITIONAL ASPECTS OF THE TRIAL

### *Histopathology*

The importance of comparability of diagnosis between each of the 8 centres was recognized, and a panel of histopathologists participating in the trial was therefore set up. The purposes of this panel are to develop an agreed common nomenclature for classifying the histology of benign and malignant breast tumours; to work towards consistency of reporting by regular reviews of certain categories of histology from women in the trial, and to study the natural history and prognostic significance of certain histological features.

### *Women's attitudes towards early-detection services*

If screening or self-examination education services are to make any sizeable impact on breast-cancer mortality, it is essential that they should be accepted and practised by the great majority of women at risk. Acceptance is influenced by women's beliefs about the causes, treatment and curability of breast cancer and their own vulnerability. The early-detection measure must be as convenient and pleasant as possible and, most importantly, must not engender undue anxiety. Sample surveys of these aspects are being conducted.

### *Radiation risks of mammography*

The 2 screening centres and 1 self-examination centre are using film-screen mammography which gives an average skin exposure around 0·2 rad. The remaining self-examination centre, which is using xerography with a tungsten target, gives an average skin exposure of 1 rad. A standardized measurement of dose is made regularly in each of the early-detection centres by a physics department independent of the trial centres.

*Economic assessment*

It is hoped to incorporate an economic assessment of the early-detection programmes after 2–3 years, when they are fully operational. This will compare the economic consequences of an early-detection policy with those of management of breast disease in the comparison centres, taking into account all follow-up costs arising from early detection.

*Studies of aetiology of breast cancer and other diseases*

The prospective follow-up of a cohort of middle-aged women with subsequent notification of incidence of breast cancer and mortality from all causes, offers an opportunity for a prospective study of various aetiological factors. Discussions are now in progress about questions or tests which might be administered to supposedly well women entering the trial, which might throw additional light on the causation of breast cancer or other diseases.

Collection of the large volume of clerical and clinical information required for this trial is made possible by the enthusiasm and dedication of a multidisciplinary team of doctors, nurses, radiographers, administrative, and especially secretarial, clerical, programming and computing staff, working in each of the 8 participating districts and the coordinating centre.

The trial as a whole is supported by grants from the Department of Health and Social Security and the Scottish Home and Health Department. The Cancer Research Campaign has contributed the additional costs of setting up and recording the effects of the breast-cancer education campaign in Centre A, and the Helen Garrod Breast Cancer Trust has contributed the costs of breast self-examination education and clinic facilities in the adjacent health district in Centre D. The Review Panel on Breast Tumour Pathology is supported by the Medical Research Council.

### REFERENCES

Bonadonna, G. (1980) Adjuvant chemotherapy of breast cancer. *Br. J. Hosp. Med.*, **1**, 40.

Chamberlain, J., Clifford, R. E., Nathan, B. E., Price, J. L. & Burn, I. (1979) Error-rates in screening for breast cancer by clinical examination and mammography. *Clin. Oncol.*, **5**, 135.

Cornstock, G. W. (1978) Uncontrolled ruminations on modern controlled trials. *Am. J. Epidemiol.*, **108**, 81.

Farquhar, J. W. (1978) The community-based model of life-style intervention trials. *Am. J. Epidemiol.*, **108**, 103.

Feinleib, M. & Zelen, M. (1969) Some pitfalls in the evaluation of screening programs. *Arch. Environ. Hlth*, **19**, 412.

Gilbertson, V. A., Church, T. R., Grewe, F. J. & 4 others (1980) The design of a study to assess occult-blood screening for colon cancer. *J. Chron. Dis.*, **33**, 107.

Kelsey, J. L. (1979) A review of the epidemiology of human breast cancer. *Epidemiol. Rev.*, **1**, 74.

Lundgren, B. (1979) Population screening by single view mammography in a geographical region. *J. Natl Cancer Inst.*, **62**, 1373.

Office of Population Censuses and Surveys (1971) *Cancer Statistics Series MB1*, 1–4.

Office of Population Censuses and Surveys (1974) *Mortality Statistics, Series DH2*, 1–5.

Patrick, D. (1980) Méthodes d'évaluation ne comportant pas de randomization: Principes et méthodes. Paper presented to *Association des Epidemiologistes de la Langue Francaise*, Paris.

Puska, P., Tuomilehta, J., Salomen, J. & 6 others (1979) Changes in coronary risk factors during comprehensive five-year community programme to control cardiovascular diseases (North Karelia Project). *Br. Med. J.*, ii, 1173.

Ramcharan, S., Cutler, J. L., Feldman, R. & 5 others (1973) Disability and chronic disease after seven years of multiphasic health check-ups. *Prevent. Med.*, **2**, 207.

Royal College of Physicians (1975) *Fluoride, Teeth and Health.* Pitman Medical.

Shapiro, S. (1978) Efficacy of breast cancer screening. In *Screening in Cancer*, UICC Technical Report, **40**, Ed. Miller. Geneva: UICC. p. 133.

Shapiro, S., Goldberg, J. & Hutchison, G. (1974) Lead time in breast cancer detection and implication for periodicity of screening. *Am. J. Epidemiol.*, **100**, 357.